# Special Issue “Role of Apoptosis and Cellular Senescence in Cancer and Aging”

**DOI:** 10.3390/ijms25042103

**Published:** 2024-02-09

**Authors:** Rumiana Tzoneva

**Affiliations:** Laboratory of Transmembrane Signaling, Institute of Biophysics and Biomedical Engineering, Bulgarian Academy of Sciences, Akad. G. Bonchev Str., bl. 21, 1113 Sofia, Bulgaria; tzoneva@bio21.bas.bg; Tel.: +359-29793242

The intention of this Special Issue is to elucidate the role of apoptosis and cellular senescence in different pathological processes, such as cancer and aging. Apoptosis and senescence are two types of cellular responses to various irreversible dysfunctions caused by cellular damage. While, in carcinogenesis, the ability of cells to trigger apoptosis and senescence progressively decreases, in aging, the accumulation of senescent cells increases, and the rate of apoptosis also significantly grows. In the early and reproductive stages of life, apoptosis and cellular senescence function as the main mechanisms of tumor suppression, providing an effective antitumor strategy, but both become destructive and promote aging later in life. In this regard, understanding the mechanisms that determine the delicate balance between apoptosis and cellular senescence will greatly contribute to the therapeutic use of both processes in the development of future anti-cancer and aging strategies, as well as the development of our understanding of “healthy aging”.

## 1. Alzheimer’s Disease, Aging

Cellular senescence occurs during the full lifespan and may be due to various stress factors, such as oncogene induction, followed by DNA damage and telomere shortening, chromatin disturbances, and inflammation [1,2]. The induction of cellular senescence and apoptosis can trigger serious alterations in the regulation of angiogenesis, and disturbing homeostasis could lead to many pathological processes, such as cancer, atherosclerosis, rheumatoid arthritis (uncontrolled cell proliferation), and neurodegenerative diseases (mitotic inhibition) [3]. Senescence and apoptosis [1] of the vascular system plays an essential role in aging and many neurodegenerative diseases, such as Alzheimer’s disease [4], Parkinson’s disease [5], and Huntington’s disease [6]. One of the main features of aging vasculature is disturbed extracellular matrix (ECM) interactions, which are a consequence of the stiffening of the ECM due to glycation, aggregation, and cross-linking [7]. The impaired ECM leads to excess angiogenesis; leaky vasculature; low shear stress; and, in the end, to non-productive angiogenesis [8]. The role of extracellular vesicles (EVs) in senescence is worthy of mentioning. For instance, since healthy cells release EVs as part of normal cellular homeostasis, senescent cells secrete EVs of pro-angiogenic molecules like HIF-1α, VEGF, MMPs, and microRNAs, which may lead to homeostasis disruption and non-productive angiogenesis and further progression of neurodegenerative diseases [9]. For instance, in AD, non-productive angiogenesis is observed around Aβ plaques, leading to the disassembly of Aβ-plaque-associated blood vessels and the phagocytic activity of microglia [10], as well as to the manifestation of cognitive disabilities. In this way, the radical scavenger melatonin was shown to play a role in minimizing non-productive angiogenesis and endothelial senescence by hindering Ab plaque formation and reducing oxidative stress (OS) [11].

Signs of senescence, except in endothelial cells, have been reported in several cells in the brains of AD patients, such as neurons [12], microglia [13], progenitor oligodendrocytes [14], neural stem cells [15], and fibroblasts due to oxygen–glucose deprivation [16]. Furthermore, there exists evidence suggesting that AD manifestations extend beyond the brain and that AD pathogenesis can be found in the peripheral tissues of patients with AD [17].

Several markers of cellular senescence, such as G0-G1 phase cell cycle arrest, p16, p53, and H2Ax activity have been found in the peripheral blood mononuclear cells (PBMCs) of amnestic mild cognitive impairment (aMCI) and AD patients [18].

## 2. Cancer

An unconventional hypothesis concerning carcinogenesis suggests that carcinogenesis occurs due to replicative senescence of epithelial cell dedifferentiation in hyperplasia, leading to mesenchymal cells with potentially cancerous behavior [19]. On the other hand, some authors believe that shifting the cancer cells from a metastable state into a more stable senescence state by matrix remodeling can help to avoid the reactivation of dormant cancer cells [20]. Nevertheless, originally senescence, was identified as an irreversible arrest of proliferation in normal cells, but lately, it has been shown that cancer cells can also undergo senescence, which can be reversible [21]. In this way, there are many concerns that senescent cancer cells might be more harmful and can result in cancer recurrence [21]. This observation should be kept in mind when using antitumor therapies.

Interestingly, epidemiological studies have led to a conclusion that the senescence process might be involved in the inverse association between cancer and AD [22]. This means that having a history of cancer protects one from the development of Alzheimer’s disease (AD), and vice versa. These phenomena can be explained by the decreased susceptibility to oxidative cell death of the peripheral blood mononuclear cells (PBMC) of amnestic cognitive impairment (aMCI) and Alzheimer’s disease (AD) patients with cancer histories. As a consequence, the cellular senescence markers of those AD or aMCI patients with cancer histories are comparable with healthy individuals.

## 3. Novel Therapeutic Strategies to Treat Cancer

This high level of tumor heterogeneity, which includes co-existence of anastatic cancer cells [23,24], drug-tolerant persister cells [23,24], oncogenic caspase 3-expressing cells [25,26,27], polyploid giant cancer cells [21], and others, impedes biomarker development for patient stratification, and also limits the therapeutic efficacy of targeted therapies and immune checkpoint inhibitors [25]. Thus, it remains critical to unravel the mechanisms promoting molecular heterogeneity in order to develop more effective biomarkers and therapeutics. 

One of the main outcomes of cancer treatment is apoptosis and/or senescence, as well as mitotic catastrophe [28]. The new data reveal the role of polyploid giant cancer cells (PGCCs) in the immortality, invasion, origin, metastasis, and resistance of tumor cells to radiotherapy and chemotherapy [21,29]. They were even proposed as a marker for chemoresistsnce and metastasis [21]. On the other hand, it has now become apparent that tumor treatment can induce senescence in cancer cells, which secrete both tumor-suppressing and tumor-promoting proteins that can serve as prognostic markers [28]. Elucidation of the regulatory mechanisms which govern both “faces” of tumor senescence makes it possible to design new therapeutic approaches to improving the efficacy and decreasing the side effects of cancer therapy. 

It is widely accepted that the hallmark of cancer is the inability of the cells to evade the mechanisms related to regulated cell death (RCD) [23]. However, the engagement of RCD does not result in cell death in all cases. Escaping cell death can lead to genomic instability and, thus, to both pro-tumor and anti-tumor immune responses [23]. Consequently, sublethal engagement of cell death could lead to metastasis, invasiveness, and unresponsiveness to therapy [23]. Apoptosis is the best described form of regulated cell death, and was, until relatively recently, considered irreversible [24]. For instance, the process of escaping apoptosis from cancer cells (anastasis) may contribute to tumor relapse following therapy, as, in some cases, cells that “recover” from apoptosis can display stem-like properties [23]. So-called apoptosis-induced proliferation is also a known compensatory mechanism by which apoptotic cells actively stimulate the neighboring non-apoptotic cancer cells to divide [26,27]. This process is mediated by apoptotic-derived extracellular vesicles which promote cancer cell proliferation and resistance to therapy [26].

Small molecule inhibitors of aurora kinases, such as VX-680, are novel, selectively acting anticancer agents currently being investigated in clinical oncology trials [30]. A long-term effect of these inhibitors has been on the proliferation of cancer cells. The data obtained from experiments on VX-680 with euploid cells point out that VX-680 elicited a G1-like arrest of cells with either 2N or 4N DNA content [30]. The majority of cells developed abnormal nuclei when exposed to VX-680 (4N DNA), underwent a permanent arrest of proliferation, and developed various phenotypes characteristic of senescence even after discontinuation of the treatment. The authors claimed that, since the polyploid state is believed to be an intermediate cell state that fuels the malignant progression of cancer cells by promoting aneuploidy, treatment with inhibitors of aurora kinases could cause these cells to undergo unproliferative arrest, which is associated with multiple morphological and molecular characteristics of senescence [30]. One of the immediate early response genes activated by different stress conditions usually causing cell senescence is Regulated in Development and DNA Damage Response 1 (REDD1)/DNA Damage-Induced Transcript 4 (DDIT4) [31]. It is thought that REDD1 plays a crucial role in cancer development because it is involved in many cell processes, such as metabolic signaling, oxidative stress, and DNA damage response. In this way, REDD1 might be a novel therapeutic target for treatment.

The induction of senescence in tumor cells is emerging as attractive approach for cancer treatment. But, at the same time, the side effect which induces senescence of cancer cells should be kept in mind, as it can cause growth stimulation of non-senescent tumor cells and/or increased pooling of de novo carcinogenesis, as well as the development of age-related diseases such as Alzheimer’s [28].
